# Bosutinib-induced palmoplantar keratoderma treated with acitretin

**DOI:** 10.1016/j.jdcr.2026.01.033

**Published:** 2026-02-02

**Authors:** Ye Chua, Katharina Wiedemeyer, Julie Mervak, Sruthi Renati

**Affiliations:** a University of Michigan Medical School, Ann Arbor, Michigan; b Department of Pathology, University of Michigan – Michigan Medicine, Ann Arbor, Michigan; c Department of Dermatology, University of Michigan – Michigan Medicine, Ann Arbor, Michigan

**Keywords:** acitretin, bosutinib, chronic myeloid leukemia, oncodermatology, palmoplantar keratoderma, tyrosine kinase inhibitor

## Introduction

Bosutinib is a second-generation tyrosine kinase inhibitor (TKI) approved by the FDA in 2012 for treatment of patients with Philadelphia chromosome-positive chronic myeloid leukemia (CML) resistant and/or intolerant to prior therapy, such as imatinib.^1^ While dermatologic adverse events (AEs) are commonly reported with bosutinib, with any grade rash occurring in 25% to 43% of patients, these typically manifest with pruritic papular and maculopapular rashes such as can be seen with morbilliform drug eruption.^2^^,^^3^ Less common cutaneous adverse drug reactions include acneiform, bullous, psoriasiform, and eczematous eruptions. Palmoplantar keratoderma (PPK) has been primarily associated with other TKIs such as imatinib, olmutinib, and sorafenib, though no reports to date have described this AE with bosutinib.^4^ We report a case of bosutinib-induced PPK in a patient with CML successfully treated with acitretin, highlighting that this cutaneous AE can be effectively managed without discontinuing essential cancer therapy.

## Case report

A 61-year-old male with CML developed progressive thickening and scaling of his palms and soles approximately 5 months after initiating bosutinib therapy, 400 mg by mouth daily.

**Fig 1:**
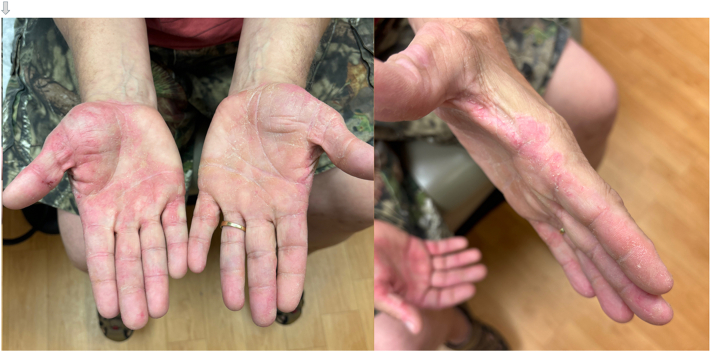
Initial presentation of diffuse palmoplantar keratoderma and erythematous lichenified scaly thin plaques of the palms extending onto lateral finger margins without dorsal involvement or nail changes.

At presentation to dermatology, physical examination revealed diffuse keratoderma and erythematous lichenified scaly thin plaques of the palms and soles extending onto the lateral hand and foot margins without dorsal involvement or nail changes (Fig 1). He reported that previous trial of multiple topical steroids had been ineffective.

**Fig 2:**
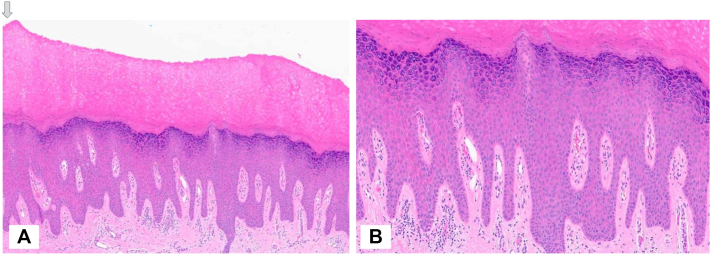
**A,** Epidermal hyperplasia with hyperparakeratosis and hypergranulosis (hematoxylin and eosin [H&E], 40×). **B,** Higher magnification shows a mild perivascular lymphocytic infiltrate within the papillary dermis (H&E, 100×).

A shave biopsy of the left index finger was performed. Histopathologic examination revealed epidermal hyperplasia with hyperparakeratosis, hypergranulosis, and a superficial perivascular lymphocytic infiltrate, compatible with PPK (Fig 2). No spongiosis or koilocytic changes were identified.

Given the temporal relationship to bosutinib therapy initiation and the histopathological findings, a diagnosis of bosutinib-induced PPK was made. The patient’s oncologist wished to continue bosutinib given excellent control of his CML. Acitretin was selected as a treatment option given that it is nonimmunosuppressive, an important consideration for a patient on cancer therapy. Baseline laboratory studies including complete blood count, comprehensive metabolic panel, and lipid panel were obtained and showed no abnormalities. Acitretin 25 mg by mouth daily and topical urea 40% cream twice daily were initiated for symptomatic management of PPK.

**Fig 3:**
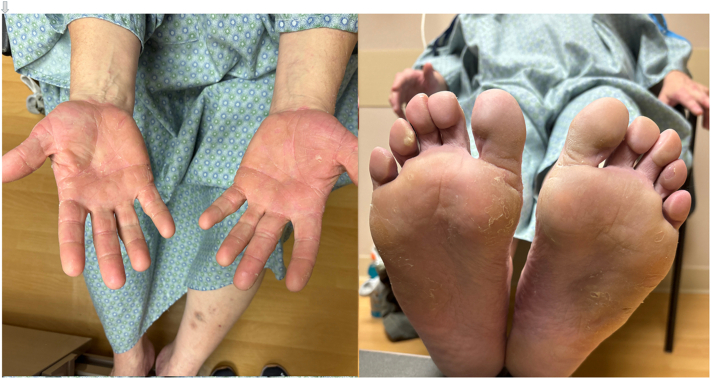
Marked reduction in erythema and hyperkeratosis of palms and soles after 6 months of acitretin treatment while bosutinib therapy was maintained.

There was minimal improvement at 3-month follow-up, and acitretin was increased to 50 mg by mouth daily. There was significant improvement in erythema and hyperkeratosis over the subsequent 4 months (Fig 3). Due to xerosis and hair thinning at the higher dose, acitretin was reduced back to 25 mg daily. At the patient’s most recent clinical evaluation (13 months after initiating acitretin), there was minimal erythema and scaling of the lateral aspects of his hands and feet with continued improvement. He remained on bosutinib throughout this time.

## Discussion

PPK is a well-recognized cutaneous AE associated with several classes of medications, including TKIs. A recent systematic review analyzing 247 patients with drug-induced PPK found that PPK was most frequently associated with BRAF inhibitors, BRAF inhibitors combined with MEK1/2 inhibitors, and TKIs.^4^ Notably, bosutinib was not mentioned in this comprehensive review. While rash is a common AE with bosutinib, reported in 25% to 43% of patients, manifestation of PPK has not been previously documented.^2^^,^^3^

However, multiple case reports have documented imatinib-induced PPK in patients with CML, with presentations ranging from isolated PPK to more extensive manifestations with nail involvement.^5^^,^^6^ Both imatinib and bosutinib are TKIs that target *BCR-ABL* pathways, and this shared molecular target may contribute to similar cutaneous manifestations observed with both agents. An alternate explanation for second generation TKIs (including bosutinib) is that inhibition of Src-family kinases may alter immune regulation and keratinocyte signaling, contributing to inflammatory and immune-related AEs, including hyperkeratotic skin changes that could manifest as PPK.^7^ Further investigation is needed to elucidate the exact mechanism underlying this uncommon AE.

A previous systematic review found that the most common treatments for drug-induced PPK were keratolytic treatments and both topical and oral corticosteroids, but complete resolution occurred in 50% of cases after discontinuation of the offending drug (mean resolution period: 2.4 months).^4^ However, in patients requiring continued TKI therapy for cancer management, discontinuation may not be feasible. Systemic retinoids, such as acitretin, have been used to manage cutaneous AEs associated with targeted cancer therapies. Acitretin exerts its therapeutic effect by binding to nuclear retinoic acid receptors, normalizing keratinocyte differentiation and reducing epidermal hyperproliferation.^8^ In this context, acitretin has been used to manage BRAF inhibitor-induced hyperkeratotic lesions and has shown benefit in some cases of imatinib-induced lichenoid eruptions.^9^^,^^10^ However, data on acitretin specifically for TKI-induced PPK remain limited. In our case, acitretin provided effective PPK control while allowing continuation of bosutinib therapy, with sustained benefit at 13 months on 25 mg maintenance dosing. This demonstrates that PPK can be managed without compromising essential cancer treatment, though long-term retinoid therapy may be necessary for patients who remain on TKIs.

This case expands the spectrum of reported cutaneous toxicities associated with bosutinib and demonstrates the importance of early dermatologic consultation when unusual skin findings develop in patients on cancer therapy. Recognition of this AE can facilitate appropriate management strategies and allow patients to effectively continue critical cancer treatments. As newer targeted treatments and immunotherapies continue to be developed and enter clinical use, dermatologists play a vital role in identifying and managing novel cutaneous AEs in collaboration with oncologists.

## Conflicts of interest

None disclosed.
